# Monthly Summary

**Published:** 1889-07

**Authors:** 


### Monthly Summary.

---

FILLING TEETH WITH VULCANITE.—While considering the properties of vulcanized rubber, it occurred to me that it would be a good material for filling labial cavities of incisor teeth. I experimented with ordinary red rubber until I became satisfied that there was nothing superior to it for the purpose, except it be porcelain. In practice I use white rubber, of which several different shades should be kept on hand in order to match the different shades of teeth. The operation is so simple that it can be explained in a few words. Shape the cavity as for an ordinary gold filling, make slight undercuts and avoiding sharp angles. Then saw off a piece of rubber, which should be vulcanized, of suitable length, say about an inch or two long, and trim the end to approximate the shape of the cavity. Line the cavity with oxyphosphate, then grasp the rubber with forceps and soften the end to be inserted in the flame of the spirit lamp and press it home. It should be held firmly in position until it cools, when it will be found that it has filled the undercuts and conformed to the shape of the cavity, and would require considerable force to dislodge it. It should then be cut off with a fine saw or separating file a little above the level of the tooth substance, and, if necessary, burnished down around the edges with a hot burnisher, and then polished off. If care is used in selecting the proper shade of rubber, you will have a filling that is almost imperceptible. When white sheet rubber is not at hand, rubber goods—of which there is a great variety—can be used; for instance, the back of a white rubber comb. Gold is too conspicuous for labial cavities of incisors, and porcelain is of necessity expensive and is limited to perfectly round cavities, while rubber is not conspicuous nor expensive and can be used for almost any shape of cavities, provided you have definite walls.—*By Morgan Adams, M. D., D. D. S. in Archives of Dentistry.*

